# (2*E*)-2-(2,4-Dichloro­phenyl­sulfon­yl)-3-(4-methyl­anilino)-3-(methyl­sulfan­yl)acrylonitrile

**DOI:** 10.1107/S1600536808012531

**Published:** 2008-05-03

**Authors:** Mario V. Capparelli, Arthur R. Barazarte, Jaime E. Charris

**Affiliations:** a Escuela de Química, Facultad de Ciencias, Universidad Central de Venezuela, Caracas 1051, Venezuela; b Facultad de Farmacia, Universidad Central de Venezuela, Caracas 1051, Venezuela

## Abstract

The title compound, C_17_H_14_Cl_2_N_2_O_2_S_2_, and the 3-methoxy­anilino analogue reported in the preceding paper have been used as starting materials to develop benzothia­zine derivatives with anti­malarial activity. The mol­ecule displays an *E* (*trans*) configuration about the central double bond. Due to conjugation in the C=C—C N group, the putative single bond shows a significant shortening [1.418 (3) Å]. The mol­ecule has a six-membered ring involving an intra­molecular N—H⋯O(sulfon­yl) bond, which is an example of resonance-assisted hydrogen bonding. In the crystal structure, bonds of the C—H⋯O(sulfon­yl) and C—H⋯N(cyano) types form double layers of mol­ecules parallel to (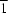
01). Within these layers there are π–π inter­actions between benzene rings of pairs of centrosymmetrically related mol­ecules, with distances of 3.7969 (12) Å between centroids. C—H⋯π interactions are also present.

## Related literature

For related literature, see: Allen (2002 ▶); Allen *et al.* (1987 ▶); Baraza­rte *et al.* (2008 ▶); Capparelli *et al.* (2008 ▶); Charris *et al.* (2005 ▶, 2007 ▶); Gilli *et al.* (1989 ▶); Hirshfeld (1976 ▶); Kennard *et al.* (2003 ▶); Krivokolysko *et al.* (2002 ▶); Song *et al.* (2005 ▶); Tominaga *et al.* (1989 ▶, 2002 ▶).
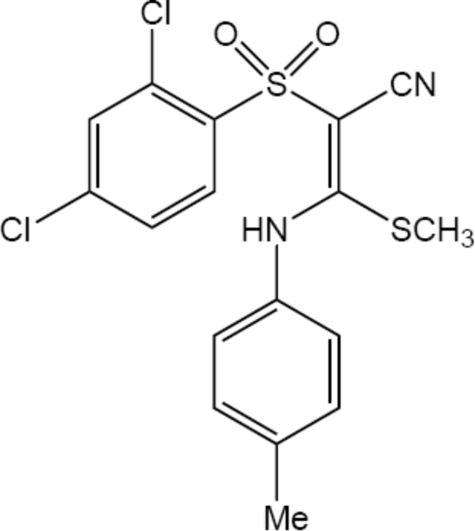


## Experimental

### 

#### Crystal data

C_17_H_14_Cl_2_N_2_O_2_S_2_
*M* *_r_* = 413.32
Monoclinic, 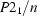

*a* = 11.3975 (6) Å
*b* = 14.8147 (8) Å
*c* = 11.7215 (6) Å
β = 109.444 (1)°
*V* = 1866.30 (17) Å^3^
*Z* = 4
Mo *K*α radiation
μ = 0.58 mm^−1^
*T* = 296 (2) K
0.55 × 0.36 × 0.27 mm

#### Data collection

Bruker SMART APEX diffractometer
Absorption correction: multi-scan (*SADABS*; Bruker, 2001 ▶) *T*_min_ = 0.765, *T*_max_ = 0.851
12685 measured reflections
4562 independent reflections
3674 reflections with *I* > 2σ(*I*)
*R*_int_ = 0.015

#### Refinement

*R*[*F* ^2^ > 2σ(*F* ^2^)] = 0.040
*wR*(*F* ^2^) = 0.117
*S* = 1.03
4562 reflections
229 parameters
2 restraints
H-atom parameters constrained
Δρ_max_ = 0.38 e Å^−3^
Δρ_min_ = −0.30 e Å^−3^

### 

Data collection: *SMART* (Bruker, 2002 ▶); cell refinement: *SAINT* (Bruker, 2001 ▶); data reduction: *SAINT*; program(s) used to solve structure: *SHELXS97* (Sheldrick, 2008 ▶); program(s) used to refine structure: *SHELXL97* (Sheldrick, 2008 ▶); molecular graphics: *ORTEP-3* (Farrugia, 1997 ▶); software used to prepare material for publication: *WinGX* (Farrugia, 1999 ▶) and *PLATON* (Spek, 2003 ▶).

## Supplementary Material

Crystal structure: contains datablocks I, global. DOI: 10.1107/S1600536808012531/bg2185sup1.cif

Structure factors: contains datablocks I. DOI: 10.1107/S1600536808012531/bg2185Isup2.hkl

Additional supplementary materials:  crystallographic information; 3D view; checkCIF report

## Figures and Tables

**Table 1:** Hydrogen-bond geometry (Å, °)

*D*—H⋯*A*	*D*—H	H⋯*A*	*D*⋯*A*	*D*—H⋯*A*
N2—H2⋯O2	0.86	2.20	2.756 (2)	123
C4—H4*A*⋯N1^i^	0.96	2.61	3.504 (3)	156
C5—H5*C*⋯O2^ii^	0.96	2.50	3.442 (3)	167
C4—H4*B*⋯*Cg*2	0.96	2.76	3.582 (3)	144

Symmetry codes: (i) 
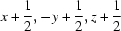
; (ii) 
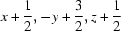
. *Cg*2 is the centroid of the C21–C26 ring.

**Fig. 1.**
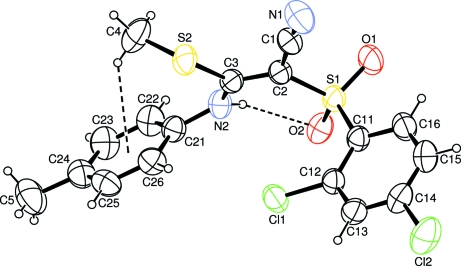
Molecular structure of (II) showing the atomic numbering. Displacement ellipsoids are drawn at 50% probability level. Possible hydrogen bonds are shown as dashed lines.

**Fig. 2.**
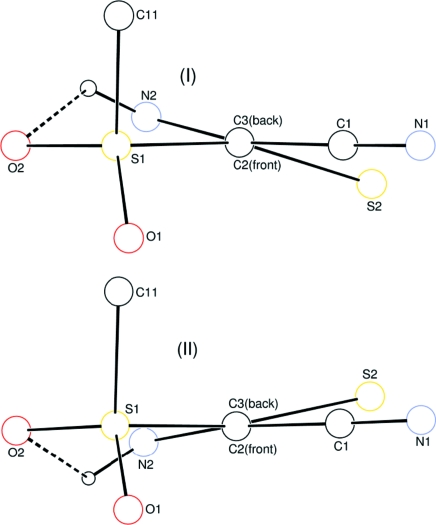
View of the molecules of (I) and (II) along the C2═C3 bonds. Possible hydrogen bonds are shown as dashed lines. For clarity, the phenyl groups (except the C11 atoms) and the S-bonded Me groups were omitted.

**Fig. 3.**
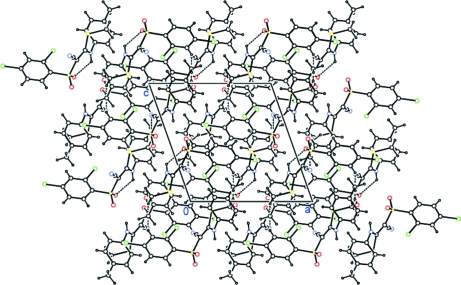
Crystal structure of (II) viewed down the b axis. Possible hydrogen bonds are shown as dashed lines.

## supplementary crystallographic information

### Comment

The exploration of simple molecules with different functionalities for the synthesis of heterocycles is a worthwhile contribution to the chemistry of these compounds. The title compound (II), and the 3''-methoxy analogue (I) [see previous paper: Capparelli *et al.*, 2008)], and similar derivatives, have been used as effective synthons in the syntheses of some 1H-pyrrole-2,5-diones (Tominaga *et al.*, 2002), pyrimidine derivatives (Tominaga *et al.*, 1989) and 4H-1,4-benzothiazine-1,1-dioxides (Charris *et al.*, 2005). We used (I) and (II) as starting materials to develop benzothiazine derivatives with antimalarial activity (Charris *et al.*, 2007; Barazarte *et al.*, 2008).

The X-ray structure determination showed that there is one molecule per asymmetric unit (Fig. 1), which displays E (*trans*) configuration about the C2═C3 double bond. A search of the Cambridge Structural Database (version 5.29, updated Jan 2008) (Allen, 2002) produced no structures with the same central fragment (*i.e.* excluding the phenyl rings) of (II) for proper comparison, but a search for the more restricted fragment *X*—C(CN)═C(SMe)-N(H)—Y gave three comparable structures, *viz*. TAKDOZ (Krivokolysko *et al.*, 2002), AJULUM (Kennard *et al.*, 2003) and DALVES (Song *et al.*, 2005). Due to conjugation in the C3═C2—C1≡N1 moiety, the putative single bond C2—C1 (1.418 (3) Å) shows a significant shortening, similar to the range 1.415 (7)–1.437 (4) Å observed in the aforementioned structures. Bond lengths (see Supplementary Materials) are in good agreement with the expected values (Allen *et al.*, 1987). Within experimental error (*i.e.* 3 e.s.d.'s) all of the corresponding bond angles and most of the bond lengths and are equal in (I) and (II).

Aside from the σ-bonded (*i.e.* free rotating) phenyl and SMe groups, the molecules of (I) and (II) display a significant difference in the rigid moieties formed by the double bonds C2═C3 and their neighboring atoms (S1, C1, S2, N2). The planes defined by S1—C2—C1 and N2—C3—S2 make dihedral angles of 17.08 (17)° in (I) and 11.2 (3)° in (II), but they are twisted about C2═ C3 in different directions (Fig. 2), probably due to packing forces.

As in (I), the title compound displays a six-membered ring involving an intramolecular N—H···O(sulfonyl) bond (Table 1), which is an example of resonance-assisted hydrogen bonding (RAHB) (Gilli *et al.*, 1989), as suggested by the ring bond lengths. Comparison with AJULUM and DALVES, which display similar rings [with N—H···O(carbonyl) and N—H···O(carboxyl) bonds respectively] and TAKDOZ, which does not have RAHB, reveal lengthenings of the C═C distances [AJULUM, 1.371 (2) Å; DALVES, 1.386 (7) Å; TAKDOZ, 1.345 (4) Å] and shortenings of the C—N bonds [AJULUM, 1.360 (2) Å; DALVES, 1.333 (6) Å; TAKDOZ, 1.404 (4) Å]. On the other hand, the bond length of S1═O2, involved in RAHB, (1.4345 (15) Å) is nearly identical to the S1═ O1 (1.4343 (15) Å). The molecular geometry also allows for a possible C4—H4b···*Cg*2 intramolecular interaction (*Cg*2: see below).

In the crystal structure (Fig. 3) bonds of the C—H···O(sulfonyl) and C—H···N(cyano) type form double layers of molecules parallel to (-1 0 1). Within these layers there are π-π interactions between phenyl rings of pairs of centrosymmetrically related molecules, with *Cg*1···*Cg*1(-*x*, 1 - *y*, -*z*), 3.7969 (12) Å (Cgm: centroid of ring Cm1—Cm6, m = 1, 2).

### Experimental

To a solution of 2,4-dichlorobenzenosulfonylacetonitrile (1.50 g, 6.1 mmol) and KOH (0.46 g, 8.1 mmol) in anhydrous dioxane (10 ml) at 0°C, was added dropwise the 4-methylphenylisothiocianate (0.91 g, 6.1 mmol) dissolved in anhydrous dioxane (3 ml). The resulting solution was stirred for 4 h at room temperature and then was added iodomethane (0.87 g, 6.1 mmol) and the mixture was stirred by 5 h at room temperature. The solvent was removed under reduced pressure and the residue was dissolved in dichloromethane (20 ml), washed with water, brine, dried over anhydrous sodium sulfate, filtered and concentrated to dryness. The solid was purified by recrystallization from ethanol. Yield: 1.91 g (74%). Crystals suitable for X-ray analysis were obtained during the recrystallization.

The IR spectrum was determined on a Shimadzu model 470 spectrophotometer. IR data [KBr pellets, ν (cm^-1^)]: 3264 (NH), 2192 (CN), 1341 (SO_2_), 1133 (SO_2_).

The ^1^H NMR spectrum was recorded using a Jeol Eclipse 270 MHz [CDCl_3_/TMS, δ (p.p.m.), atomic numbering as in Fig. 1]: 2.20 (s, 3H, SCH3), 2.37 (s, 3H, CH_3_), 7.13 (d, 2H, H22, H26; J: 8.3 Hz), 7.21 (d, 2H, H23, H25; J: 8.3 Hz), 7.46 (dd, 1H, H15; J: 8.6, 1.9 Hz), 7.56 (d, 1H, H13; J: 1.9 Hz), 8.14 (d, 1H, H16; J: 8.6 Hz), 9.90 (brs, 1H, NH).

The elemental analysis was performed on a Perkin Elmer 2400 CHN analyzer; results (%) were within ±0.4 of predicted values. Calculated for C_17_H_14_N_2_O_2_Cl_2_S_2_: C, 49.40; H, 3.41; N, 6.78. Found: C, 49.35; H, 3.40; N, 6.83.

The melting point (uncorrected) was measured with a Fischer-Johns micro hot-stage apparatus: 182–184 °C.

### Refinement

Hydrogen atoms were placed in calculated positions using a riding atom model with fixed C—H distances [0.86 Å for N, 0.93 Å for C(*sp*^2^), 0.96 Å for C(*sp*^3^)] and *U*_iso_ = p *U*_eq_(parent atom) [p = 1.2 for N and C(*sp*^2^), 1.5 for C(*sp*^3^)].

DELU restraints (Sheldrick, 2008) were applied to C1—C2 and S1—O2 bonds which (before restraints) had values of ΔU/σ = 6.00 Å and 5.13 Å, somewhat above the standard maximun (*i.e.* ΔU/σ≤ 5 Å, Spek, 1998) for the rigid-bond test (Hirshfeld, 1976).

### Crystal data

**Table:**

C_17_H_14_Cl_2_N_2_O_2_S_2_	*F*_000_ = 848
*M**_r_* = 413.32	*D*_x_ = 1.471 Mg m^−^^3^
Monoclinic, *P*2_1_/*n*	Melting point = 455–457 K
Hall symbol: -P 2yn	Mo *K*α radiation λ = 0.71073 Å
*a* = 11.3975 (6) Å	Cell parameters from 5367 reflections
*b* = 14.8147 (8) Å	θ = 2.3–28.5º
*c* = 11.7215 (6) Å	µ = 0.59 mm^−^^1^
β = 109.444 (1)º	*T* = 296 (2) K
*V* = 1866.30 (17) Å^3^	Prism, colorless
*Z* = 4	0.55 × 0.36 × 0.27 mm

### Data collection

**Table:**

Bruker SMART APEX diffractometer	4562 independent reflections
Radiation source: fine-focus sealed tube	3674 reflections with *I* > 2σ(*I*)
Monochromator: graphite	*R*_int_ = 0.015
Detector resolution: 8.13 pixels mm^-1^	θ_max_ = 29.0º
*T* = 296(2) K	θ_min_ = 2.2º
φ and ω scans	*h* = −14→12
Absorption correction: multi-scan(SADABS; Bruker, 2001)	*k* = −19→19
*T*_min_ = 0.765, *T*_max_ = 0.851	*l* = −15→14
12685 measured reflections	

### Refinement

**Table:**

Refinement on *F*^2^	Secondary atom site location: difference Fourier map
Least-squares matrix: full	Hydrogen site location: inferred from neighbouring sites
*R*[*F*^2^ > 2σ(*F*^2^)] = 0.040	H-atom parameters constrained
*wR*(*F*^2^) = 0.117	*w* = 1/[σ^2^(*F*_o_^2^) + (0.0628*P*)^2^ + 0.5083*P*] where *P* = (*F*_o_^2^ + 2*F*_c_^2^)/3
*S* = 1.03	(Δ/σ)_max_ = 0.001
4562 reflections	Δρ_max_ = 0.38 e Å^−^^3^
229 parameters	Δρ_min_ = −0.30 e Å^−^^3^
2 restraints	Extinction correction: none
Primary atom site location: structure-invariant direct methods	

### Special details

**Table:**

Geometry. All e.s.d.'s (except the e.s.d. in the dihedral angle between two l.s. planes) are estimated using the full covariance matrix. The cell e.s.d.'s are taken into account individually in the estimation of e.s.d.'s in distances, angles and torsion angles; correlations between e.s.d.'s in cell parameters are only used when they are defined by crystal symmetry. An approximate (isotropic) treatment of cell e.s.d.'s is used for estimating e.s.d.'s involving l.s. planes.
Refinement. Refinement of *F*^2^ against ALL reflections. The weighted *R*-factor *wR* and goodness of fit *S* are based on *F*^2^, conventional *R*-factors *R* are based on *F*, with *F* set to zero for negative *F*^2^. The threshold expression of *F*^2^ > σ(*F*^2^) is used only for calculating *R*-factors(gt) *etc*. and is not relevant to the choice of reflections for refinement. *R*-factors based on *F*^2^ are statistically about twice as large as those based on *F*, and *R*- factors based on ALL data will be even larger.

### Fractional atomic coordinates and isotropic or equivalent isotropic displacement parameters (Å^2^)

**Table:**

	*x*	*y*	*z*	*U*_iso_*/*U*_eq_	
S1	0.36812 (4)	0.42470 (4)	0.06767 (4)	0.04761 (14)	
S2	0.64407 (5)	0.37305 (4)	0.41840 (4)	0.05631 (15)	
Cl1	0.30933 (5)	0.54484 (4)	0.27681 (5)	0.05801 (15)	
Cl2	−0.14871 (5)	0.40783 (6)	0.14708 (6)	0.0824 (2)	
O1	0.35547 (15)	0.36287 (12)	−0.02987 (13)	0.0681 (4)	
O2	0.40074 (13)	0.51660 (10)	0.05390 (12)	0.0553 (3)	
N1	0.4303 (2)	0.21530 (14)	0.23381 (18)	0.0737 (6)	
N2	0.60865 (15)	0.51086 (11)	0.25871 (13)	0.0494 (4)	
H2	0.5904	0.5266	0.1842	0.059*	
C11	0.22335 (17)	0.42192 (12)	0.09442 (15)	0.0438 (4)	
C12	0.19883 (17)	0.47436 (12)	0.18235 (16)	0.0445 (4)	
C13	0.08383 (18)	0.47058 (14)	0.19803 (18)	0.0522 (4)	
H13	0.0672	0.5062	0.2562	0.063*	
C14	−0.00560 (18)	0.41325 (15)	0.12618 (18)	0.0542 (5)	
C15	0.0156 (2)	0.36108 (15)	0.0379 (2)	0.0610 (5)	
H15	−0.0462	0.3234	−0.0105	0.073*	
C16	0.1306 (2)	0.36578 (14)	0.02256 (18)	0.0554 (5)	
H16	0.1460	0.3308	−0.0368	0.066*	
C21	0.67534 (18)	0.57496 (13)	0.34797 (16)	0.0466 (4)	
C22	0.7764 (2)	0.61972 (15)	0.33448 (19)	0.0577 (5)	
H22	0.7990	0.6098	0.2662	0.061 (6)*	
C23	0.8437 (2)	0.67925 (16)	0.4231 (2)	0.0639 (6)	
H23	0.9115	0.7092	0.4133	0.077*	
C24	0.8129 (2)	0.69545 (14)	0.5260 (2)	0.0586 (5)	
C25	0.7089 (2)	0.65227 (14)	0.53511 (19)	0.0583 (5)	
H25	0.6848	0.6634	0.6022	0.070*	
C26	0.6398 (2)	0.59308 (14)	0.44742 (19)	0.0534 (5)	
H26	0.5695	0.5655	0.4553	0.064*	
C1	0.45350 (19)	0.28900 (14)	0.22053 (17)	0.0524 (4)	
C2	0.47563 (17)	0.38061 (13)	0.19901 (15)	0.0453 (4)	
C3	0.57197 (17)	0.42866 (13)	0.28006 (15)	0.0438 (4)	
C4	0.8069 (2)	0.3985 (2)	0.4575 (2)	0.0769 (7)	
H4A	0.8543	0.3577	0.5193	0.115*	
H4B	0.8219	0.4594	0.4869	0.115*	
H4C	0.8316	0.3922	0.3872	0.115*	
C5	0.8896 (3)	0.75847 (17)	0.6233 (3)	0.0873 (8)	
H5A	0.9753	0.7403	0.6484	0.131*	
H5B	0.8605	0.7563	0.6912	0.131*	
H5C	0.8819	0.8189	0.5921	0.131*	

### Atomic displacement parameters (Å^2^)

**Table:**

	*U*^11^	*U*^22^	*U*^33^	*U*^12^	*U*^13^	*U*^23^
S1	0.0439 (2)	0.0669 (3)	0.0322 (2)	0.0030 (2)	0.01291 (17)	−0.00344 (19)
S2	0.0637 (3)	0.0598 (3)	0.0387 (2)	−0.0013 (2)	0.0081 (2)	0.0079 (2)
Cl1	0.0546 (3)	0.0655 (3)	0.0544 (3)	−0.0084 (2)	0.0188 (2)	−0.0189 (2)
Cl2	0.0437 (3)	0.1218 (6)	0.0828 (4)	−0.0037 (3)	0.0227 (3)	0.0103 (4)
O1	0.0607 (9)	0.1015 (12)	0.0413 (7)	0.0062 (8)	0.0161 (6)	−0.0211 (7)
O2	0.0475 (7)	0.0724 (9)	0.0458 (7)	−0.0004 (6)	0.0153 (6)	0.0141 (6)
N1	0.0953 (15)	0.0615 (12)	0.0611 (11)	−0.0123 (11)	0.0217 (11)	−0.0057 (9)
N2	0.0524 (9)	0.0587 (9)	0.0349 (7)	−0.0031 (7)	0.0116 (6)	0.0047 (6)
C11	0.0420 (9)	0.0517 (10)	0.0366 (8)	0.0000 (7)	0.0115 (7)	0.0002 (7)
C12	0.0428 (9)	0.0503 (10)	0.0379 (8)	−0.0005 (7)	0.0100 (7)	−0.0004 (7)
C13	0.0476 (10)	0.0653 (12)	0.0455 (10)	0.0058 (9)	0.0177 (8)	0.0005 (9)
C14	0.0409 (10)	0.0707 (13)	0.0496 (10)	0.0016 (9)	0.0132 (8)	0.0121 (9)
C15	0.0523 (12)	0.0683 (13)	0.0556 (12)	−0.0123 (10)	0.0088 (9)	−0.0044 (10)
C16	0.0544 (11)	0.0624 (12)	0.0465 (10)	−0.0041 (9)	0.0130 (9)	−0.0104 (9)
C21	0.0468 (10)	0.0504 (10)	0.0412 (9)	0.0003 (8)	0.0129 (7)	0.0037 (7)
C22	0.0588 (12)	0.0696 (13)	0.0495 (11)	−0.0061 (10)	0.0243 (9)	0.0057 (9)
C23	0.0618 (13)	0.0639 (13)	0.0672 (13)	−0.0165 (10)	0.0228 (11)	0.0040 (10)
C24	0.0699 (13)	0.0439 (10)	0.0570 (11)	−0.0023 (9)	0.0145 (10)	0.0035 (9)
C25	0.0749 (14)	0.0521 (11)	0.0532 (11)	0.0017 (10)	0.0282 (10)	−0.0035 (9)
C26	0.0534 (11)	0.0566 (11)	0.0554 (11)	−0.0028 (9)	0.0251 (9)	−0.0004 (9)
C1	0.0586 (12)	0.0573 (10)	0.0413 (9)	0.0000 (9)	0.0167 (8)	−0.0073 (8)
C2	0.0472 (10)	0.0520 (9)	0.0358 (8)	0.0051 (8)	0.0128 (7)	−0.0026 (7)
C3	0.0445 (9)	0.0543 (10)	0.0334 (8)	0.0070 (8)	0.0139 (7)	0.0004 (7)
C4	0.0582 (14)	0.1022 (19)	0.0608 (14)	0.0158 (13)	0.0070 (11)	0.0216 (13)
C5	0.112 (2)	0.0604 (14)	0.0782 (17)	−0.0191 (14)	0.0166 (16)	−0.0122 (12)

### Geometric parameters (Å, °)

**Table:**

S1—O1	1.4343 (15)	C16—H16	0.9300
S1—O2	1.4345 (15)	C21—C26	1.382 (3)
S1—C2	1.7442 (18)	C21—C22	1.383 (3)
S1—C11	1.7796 (19)	C22—C23	1.384 (3)
S2—C3	1.7596 (18)	C22—H22	0.9300
S2—C4	1.798 (3)	C23—C24	1.385 (3)
Cl1—C12	1.7245 (18)	C23—H23	0.9300
Cl2—C14	1.730 (2)	C24—C25	1.382 (3)
N1—C1	1.146 (3)	C24—C5	1.508 (3)
N2—C3	1.338 (2)	C25—C26	1.381 (3)
N2—C21	1.430 (2)	C25—H25	0.9300
N2—H2	0.8600	C26—H26	0.9300
C11—C16	1.388 (3)	C1—C2	1.418 (3)
C11—C12	1.391 (3)	C2—C3	1.386 (3)
C12—C13	1.384 (3)	C4—H4A	0.9600
C13—C14	1.378 (3)	C4—H4B	0.9600
C13—H13	0.9300	C4—H4C	0.9600
C14—C15	1.375 (3)	C5—H5A	0.9600
C15—C16	1.383 (3)	C5—H5B	0.9600
C15—H15	0.9300	C5—H5C	0.9600
			
O1—S1—O2	118.52 (10)	C23—C22—H22	120.2
O1—S1—C2	108.61 (9)	C22—C23—C24	121.7 (2)
O2—S1—C2	108.81 (9)	C22—C23—H23	119.1
O1—S1—C11	105.82 (9)	C24—C23—H23	119.1
O2—S1—C11	109.39 (9)	C25—C24—C23	117.4 (2)
C2—S1—C11	104.83 (9)	C25—C24—C5	121.6 (2)
C3—S2—C4	105.22 (10)	C23—C24—C5	121.0 (2)
C3—N2—C21	126.20 (15)	C26—C25—C24	121.8 (2)
C3—N2—H2	116.9	C26—C25—H25	119.1
C21—N2—H2	116.9	C24—C25—H25	119.1
C16—C11—C12	118.95 (17)	C25—C26—C21	119.76 (19)
C16—C11—S1	118.07 (14)	C25—C26—H26	120.1
C12—C11—S1	122.97 (14)	C21—C26—H26	120.1
C13—C12—C11	120.56 (17)	N1—C1—C2	176.8 (2)
C13—C12—Cl1	117.44 (14)	C3—C2—C1	121.12 (17)
C11—C12—Cl1	122.00 (14)	C3—C2—S1	125.19 (14)
C14—C13—C12	118.97 (18)	C1—C2—S1	113.64 (14)
C14—C13—H13	120.5	N2—C3—C2	124.40 (16)
C12—C13—H13	120.5	N2—C3—S2	121.29 (14)
C15—C14—C13	121.78 (19)	C2—C3—S2	114.31 (14)
C15—C14—Cl2	119.36 (17)	S2—C4—H4A	109.5
C13—C14—Cl2	118.85 (16)	S2—C4—H4B	109.5
C14—C15—C16	118.76 (19)	H4A—C4—H4B	109.5
C14—C15—H15	120.6	S2—C4—H4C	109.5
C16—C15—H15	120.6	H4A—C4—H4C	109.5
C15—C16—C11	120.96 (19)	H4B—C4—H4C	109.5
C15—C16—H16	119.5	C24—C5—H5A	109.5
C11—C16—H16	119.5	C24—C5—H5B	109.5
C26—C21—C22	119.52 (19)	H5A—C5—H5B	109.5
C26—C21—N2	120.82 (17)	C24—C5—H5C	109.5
C22—C21—N2	119.66 (17)	H5A—C5—H5C	109.5
C21—C22—C23	119.66 (19)	H5B—C5—H5C	109.5
C21—C22—H22	120.2		
			
O1—S1—C11—C16	−0.27 (19)	C21—C22—C23—C24	0.1 (4)
O2—S1—C11—C16	128.48 (16)	C22—C23—C24—C25	−2.3 (3)
C2—S1—C11—C16	−114.99 (16)	C22—C23—C24—C5	178.2 (2)
O1—S1—C11—C12	−179.20 (16)	C23—C24—C25—C26	1.9 (3)
O2—S1—C11—C12	−50.45 (17)	C5—C24—C25—C26	−178.7 (2)
C2—S1—C11—C12	66.08 (17)	C24—C25—C26—C21	0.8 (3)
C16—C11—C12—C13	0.1 (3)	C22—C21—C26—C25	−3.1 (3)
S1—C11—C12—C13	178.98 (15)	N2—C21—C26—C25	177.15 (18)
C16—C11—C12—Cl1	179.16 (15)	O1—S1—C2—C3	133.90 (16)
S1—C11—C12—Cl1	−1.9 (2)	O2—S1—C2—C3	3.58 (19)
C11—C12—C13—C14	0.8 (3)	C11—S1—C2—C3	−113.35 (17)
Cl1—C12—C13—C14	−178.37 (15)	O1—S1—C2—C1	−48.61 (17)
C12—C13—C14—C15	−1.3 (3)	O2—S1—C2—C1	−178.93 (14)
C12—C13—C14—Cl2	179.34 (15)	C11—S1—C2—C1	64.14 (15)
C13—C14—C15—C16	0.9 (3)	C21—N2—C3—C2	157.68 (18)
Cl2—C14—C15—C16	−179.72 (17)	C21—N2—C3—S2	−21.5 (3)
C14—C15—C16—C11	0.0 (3)	C1—C2—C3—N2	170.85 (18)
C12—C11—C16—C15	−0.4 (3)	S1—C2—C3—N2	−11.8 (3)
S1—C11—C16—C15	−179.42 (17)	C1—C2—C3—S2	−9.9 (2)
C3—N2—C21—C26	−46.0 (3)	S1—C2—C3—S2	167.40 (11)
C3—N2—C21—C22	134.3 (2)	C4—S2—C3—N2	−39.06 (19)
C26—C21—C22—C23	2.6 (3)	C4—S2—C3—C2	141.68 (16)
N2—C21—C22—C23	−177.62 (19)		

### Hydrogen-bond geometry (Å, °)

**Table:**

*D*—H···*A*	*D*—H	H···*A*	*D*···*A*	*D*—H···*A*
N2—H2···O2	0.86	2.20	2.756 (2)	123
C4—H4A···N1^i^	0.96	2.61	3.504 (3)	156
C5—H5C···O2^ii^	0.96	2.50	3.442 (3)	167
C16—H16···O1	0.93	2.41	2.828 (3)	107
C4—H4B···Cg2	0.96	2.76	3.582 (3)	144

Symmetry codes: (i) *x*+1/2, −*y*+1/2, *z*+1/2; (ii) *x*+1/2, −*y*+3/2, *z*+1/2.
